# Toluene: correlation between occupational exposure limits and biological exposure indices

**DOI:** 10.47626/1679-4435-2022-715

**Published:** 2023-02-13

**Authors:** Aldrin Robson Lima

**Affiliations:** 1 Toximaster Higiene e Toxicologia - Avaliações Ambientais, São Paulo, SP, Brazil

**Keywords:** toluene, biomarkers, occupational medicine, occupational health, occupational exposure limit, tolueno, indicadores biológicos, medicina do trabalho, saúde do trabalhador, limite de exposição ocupacional

## Abstract

Threshold limit values for chemical substances and biological exposure indices are the main tools used in occupational hygiene and occupational medicine to control worker exposure levels. The correlation between these limits and indicators is of fundamental importance. The setting of new toluene exposure limits has raised discussion about which indicator to use. This article aims to enrich this debate with scientific data. Through a literature review, we provide a broad analysis of the factors that led to the lowering of the occupational exposure limit. Although internationally, biological indicators for toluene were replaced more than a decade ago, Brazilian authorities only began to discuss changing them in 2020. Toluene is a concern due to critical effects observed in exposed individuals, especially miscarriage. Urinary οrtho-cresol was suggested as the main biomarker in 2007. Given the broad data analysis, there are no doubts about the utility of οrtho-cresol as a biological indicator for toluene; what is lacking now is implementation of a monitoring system to comply with the legislation.

## Abstract (pt)

Os limites de exposição ocupacional às substâncias químicas e os indicadores biológicos de exposição são as principais ferramentas usadas pela Higiene Ocupacional e Medicina do Trabalho para o controle de exposições dos trabalhadores, e a correlação entre esses parâmetros é de fundamental importância. A adoção de novos limites de exposição ao tolueno abriu o debate sobre qual indicador usar, e este artigo tem como objetivo trazer dados científicos para essa discussão. Através de revisão bibliográfica, foi feita uma análise ampla dos fatores que levaram à redução do limite de exposição ocupacional. Embora, no cenário internacional, a substituição dos indicadores biológicos para o tolueno tenha acontecido há mais de uma década, somente no ano de 2020 as autoridades brasileiras trouxeram à tona essa discussão, com a alteração do indicador biológico de referência na Norma Regulamentadora n.º 7. A preocupação em torno do tolueno está baseada em seus efeitos críticos observados em indivíduos expostos, especialmente a indução de aborto espontâneo. Nesse sentido, já em 2007 foi sugerido o οrto-cresol urinário como principal biomarcador. Diante da ampla análise dos dados, não há dúvidas da representatividade do οrto-cresol como indicador biológico para o tolueno, restando agora a implementação de uma sistemática de monitorização para atender à legislação.

## INTRODUCTION

Toluene’s uses are extremely diversified, including as a component in solvents, varnishes, thinners, and cleaning liquids. It is also used industrially in the production of rubber, plastics, and asphalt derivatives. It is also used as a raw material in various syntheses, including toluene diisocyanate, dynamite, adhesives, and glues.^1^ Although used in a wide range of production processes, it was in the printing industry, especially rotogravure, that its effects on the health a large number of workers were noticed.^2^ Another reason for researching toluene, including studies on toxicokinetics, toxicodynamics, and critical short and long term effects, is its use as a drug of abuse.^3^

In its 2006 annual guide to threshold limit values (TLV) and biological exposure indices, the American Conference of Governmental Industrial Hygienists (ACGIH), which guides occupational hygiene (including exposure to chemical agents), added a note of intent to significantly change the TLV of toluene based on well-founded studies, mainly in the rotogravure industry. In 2007, the proposal was adopted, and the ACGIH recommended reducing the TLV from 50 to 20 ppm for an 8-hour workday and a 40-hour workweek.^4^ Although this change is challenging for companies that use toluene in their production processes, the publication was well received by worker health professionals, who considered the limit adequate in view of the toxic effects of toluene; it is teratogenic, reproductive, hepatotoxic, nephropathic, ototoxic, affects the ability to distinguish colors, and is suspected of causing degeneration of the myelin sheath in the peripheral nervous system.^5^

The next step towards new standards was discussion of biological exposure indices and their correlation with the revised TLV. In 2007, the ACGIH recommended the following biomarkers as biological exposure indices: urinary hippuric acid (HA-u), blood toluene, and urinary ο-cresol. In 2010, urinary toluene (TOL-u) was included as a biological exposure index and HA-u was definitively removed.^4^ Until that point in Brazil, this sector had been regulated by the Ministry of Labor through regulatory norms. In ordinance 3214 (July 8, 1978), which included the biological indicators in Table I of Regulatory Norm 7, only HA-u was determined to be a biological indicator for toluene, and this remained in force for another 10 years.^6^

In 2020, a revision of Regulatory Norm 7 was published by the Ministry of the Economy’s Special Secretariat for Social Security and Labor (Secretaria Especial de Previdência e Trabalho do Ministério da Economia), the agency that replaced the Ministry of Labor, and Table I of Annex I determined that urinary ο-cresol, blood toluene, and TOL-u should be used as biological indicators for toluene, in line with current ACGIH recommendations. Although Brazilian legislation is now in line with international recommendations, it is necessary to understand what each of these biological indicators means in practice (based mainly on their toxicokinetics), how Brazilian laboratories will meet this new demand and, especially, how this issue will be dealt with in company worker health programs.^7^

## METHODS

This study’s methodology was a bibliographic review of books and periodicals, especially ACGIH yearbooks from 1998 to 2021. National and international electronic databases featuring publications on occupational hygiene and toxicological analysis were also used, with no date limits set for articles. The search terms included “tolueno”, “tolueno toxicocinética”, “tolueno toxicodinâmica”, “ACGIH”, “National Institute for Occupational Safety and Health” (NIOSH), “tolueno métodos analíticos”, “bioindicadores”, “tolueno abuso”, “ο-cresol”, “ácido hipúrico”, “tolueno higiene ocupacional”, as well as “e/ou” and “and/or”. Research began in August 2019 and ended in October 2021. The guiding question for this review was: “What is the correlation between TLVs and biological indicators for toluene?”

## RESULTS

### THE MOST IMPORTANT PHYSICOCHEMICAL CHARACTERISTICS OF TOLUENE

Toluene (CAS 108-88-3) is an aromatic compound now obtained from petroleum, tar distillation, and mineral coal. One of its first sources was balsam of the Tolu tree (*Myroxylon toluiferum Kunth*), hence the name toluene. At room temperature, it is a colorless liquid, insoluble in water and highly volatile. Methylbenzene, tol, toluol, tolusol and phenylmethane are synonyms. The chemical properties of toluene include: explosiveness of 1.4 to 6.7% in air, significant lipid solubility, water solubility of 0.063 g/100 g at 25ºC, high flammability, characteristic odor of aromatic compounds, an olfactory threshold of 2.14 ppm (8 mg/m^3^), a boiling point of 110.6°C (760 mmHg), a melting point of 94.9°C, self-ignition at 480°C, a density of 0.867 at 25°C, vapor pressure of 28.4 mmHg at 25°C (3.8 kPa), and a molecular weight of 92.14.^8^

### TOXICOKINETICS OF TOLUENE

Individual variability can strongly influence the toxicokinetics of toluene, including factors such as heart rate, body fat volume, diet, and circadian cycle, in addition to interaction with salicylates, ethanol, and other solvents, such as n-hexane, benzene, xylenes, methyl ethyl ketone, 2-propanol and methanol. Environmental factors can also interfere in this process.^5^

### ABSORPTION

The main route of occupational exposure is inhalation, although toluene can also be absorbed through the skin. Absorption by ingestion is almost complete, although this rarely occurs in occupational settings. The lungs rapidly absorb inhaled toluene (approximately 40 to 50%), reaching peak serum concentrations within 15 to 30 minutes of inhalation, with the first 10 minutes of exposure being the most critical phase.^9^ Toluene concentrations can vary, with an accelerated rise in blood concentration followed by a fall due to lipid binding, followed by a subsequent rise as it passes back into the bloodstream. Absorption through the skin occurs while toluene is still in the liquid phase. Due to its high volatility, absorption by this route is low, but quite fast due to the compound’s high liposolubility. It should also be considered that workers whose activities involve physical exertion have shown higher absorption of toluene.^9^

### DISTRIBUTION

Toluene easily passes through cell membranes and is distributed mainly in adipose and highly vascularized tissues, such as the brain and white matter, bone marrow, liver, kidneys, and nerve tissue, which makes it difficult to measure exact blood levels. The biological half-life of toluene is 3 min in highly vascularized organs and approximately 40 min in soft tissues (eg, muscles). In adipose tissue and bone marrow, its biological half-life is approximately 738 minutes.^8,9^

### BIOTRANSFORMATION

**Figure 1:**
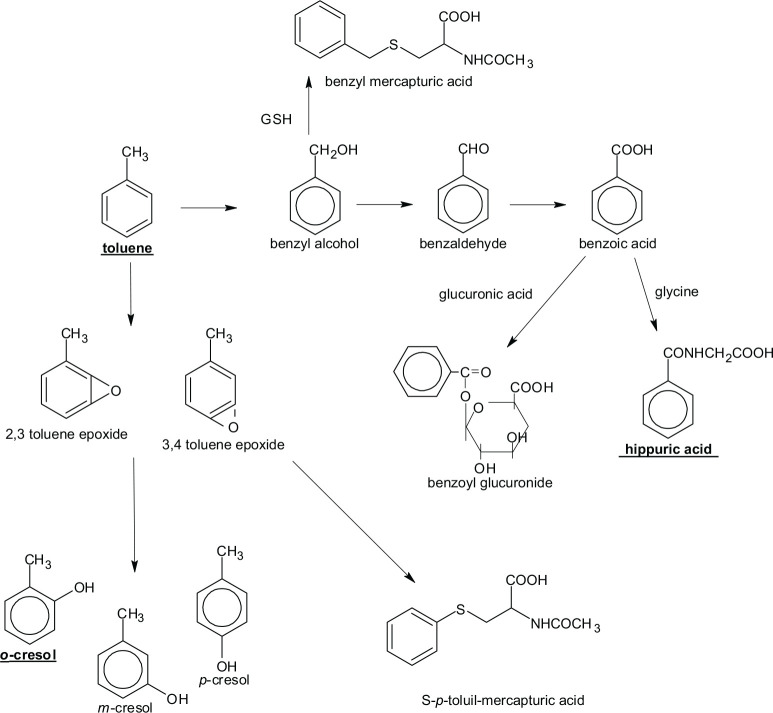
Main pathways of toluene biotransformation.

As can be seen in Figure 1, toluene has two main pathways of biotransformation: toluene epoxide and benzyl alcohol. Toluene is oxidized to epoxide compounds, which give rise to cresols (ortho-, meta , and para-) through benzene ring hydroxylation by enzymes CYP1A2, CYP2E1 and CYP2B6^9^. On the other hand, toluene can undergo hydroxylation from the methyl group to form benzyl alcohol, mediated by enzymes of the cytochrome P450 system (CYP2E1, CYP2B6, CYP2C8 and CYP1A2). Hydroxylation of the methyl group by CYP2E1 occurs in approximately 80% of absorbed toluene. In the second phase, benzyl alcohol is oxidized in two steps to benzoic acid by alcohol dehydrogenase and aldehyde dehydrogenase. Finally, it combines with the amino acid glycine, giving rise to hippuric acid. The formation of S-benzylmercapturic acid, resulting from the conjugation of benzyl alcohol and S-p-toluylmercapturic acid, is due to the conjugation of 3,4-toluene epoxide with glutathione. Although it is excreted in small amounts in the urine, it is a potential biomarker of toluene.^8,9^

### ELIMINATION

Most toluene is eliminated as HA-u (31-80%) within 20 hours of exposure. Approximately 7-14% of toluene is excreted intact through expiration. Less than 2% is excreted in bile. Phenolic and mercapturic metabolites are excreted in small proportions, approximately 1% p-cresol, 0.1% ο-cresol, and < 2% S-benzylmercapturic and S-ρ-toluylmercapturic acids are found in urine.^10^

### THE TOXICODYNAMICS AND CRITICAL EFFECTS OF TOLUENE

#### Neurotoxicity

Central nervous system dysfunction is a critical health concern following acute, intermediate, or chronic exposure to toluene, primarily via inhalation. Its mechanisms of action are mostly membrane and membrane channel changes, leading to effects such as nervous system depression and narcosis.^5,8^ The region most affected by toluene is the cerebellum. Even at exposures to low concentrations (49-130 ppm), loss of manual dexterity, verbal memory, and visual ability (including loss of color distinction) have been observed. In more severe chronic exposure, cerebral and cortical degeneration, deafness, peripheral neuropathy, and optic atrophy, including evidence of hypothalamic dysfunction, have been observed. Some of this damage may be irreversible.^8^

Toluene’s mechanism of toxicity is still not clear; few in-depth studies have been conducted on the subject and there are many gaps in the experimental models. Some scholars consider toluene’s effects similar to those of other depressants, such as benzodiazepines, barbiturates and ethanol. It is understood that the mechanism of change in neuron membrane function involves the fluidization of cell membranes, altering their permeability and function. However, the molecular aspects of this fluidization are still unclear. The dissolution of lipid layers in cell membranes and myelin explains the long-term effects, including atrophied brain regions and changes in nerve transmission. Toluene binds to the gamma-aminobutyric acid-A receptor-benzodiazepine-chlorine channel complex. Thus, there is an influx of chlorine through the membrane, leading to neuronal hyperpolarization, which inhibits nerve impulse propagation, depressing the central nervous system.^8,9^

In animal experiments, exposure to toluene has been associated with significantly higher concentrations of catecholamine neurotransmitters and their metabolites in various brain regions. As a drug of abuse, toluene’s toxicological effects similar to those of barbiturates and ethanol, causing altered dopamine levels in the brain.^11^

#### Genotoxicity

The genotoxic or mutagenic action of toluene includes sudden transmissible alteration of the genetic material. Exposure to certain agents can increase the occurrence of mutations. Many can directly interfere with DNA bases or form complexes that hinder replication.^12^ Although several studies have suggested that toluene has genotoxic potential, some authors do not consider it a mutagenic agent, since no relationship has been proven between exposure and chromosomal aberrations in animal studies.^8^

#### Reproductive toxicity

The mammalian reproduction process involves several stages, from sexual maturation to the birth of new individuals. During gestation, maternal exposure to a toxic agent can lead to different responses, from teratogenicity to death of the embryo or fetus, with the greatest risk occurring in first 12 weeks after conception.^13^

Regarding fetotoxicity, the mechanisms by which toluene decreases fetal development (even leading to miscarriage) have not yet been elucidated. The study that gave rise to the revised TLV demonstrated that toluene has embryotoxic, fetotoxic, and teratogenic properties in rat and rabbit models. Studies on occupational exposure to toluene in pregnant women in the gravure printing industry have confirmed cases of children born with central nervous system disorders, organ, brain, and limb anomalies and growth retardation.^2^ Another animal experiment found that acetylsalicylic acid dramatically potentiates these properties,^14^ corroborating the results of studies on the interference of salicylates in toluene toxicokinetics.^9^

#### Other adverse effects

Exposure to toluene can irritate the mucous membrane of the respiratory tract, and severe exposure can lead to fluid build-up in the lungs and even pneumonia. Exposure may cause bronchospasms in people with asthma or chronic obstructive pulmonary disease. Toluene is a strong skin irritant since, due to its high liposolubility, it removes natural lipids from the upper layers, leading to contact dermatitis.^15^ Toluene is also ototoxic in cases of chronic exposure to high concentrations. In 1977, a study by the University of São Paulo and NIOSH showed that noise had an additive effect on exposure to toluene below the then-current TLV.^3^ Azevedo^16^ reported on the ototoxic effects of toluene in association with noise exposure, particularly that toluene can potentiate hearing loss. An additive effect was also observed with concomitant acetylsalicylic acid use.^16^

Chronic exposure to toluene above the TLV can cause hepatotoxicity and nephrotoxicity, leading to proteinuria, as well as lesions in the glomeruli and renal tubules.^8^

### ANALYTICAL DETERMINATION OF OCCUPATIONAL EXPOSURE INDICATORS

#### Environmental monitoring

Environmental monitoring is the periodic assessment of occupational exposure by measuring the concentration of chemical agents in the workplace, followed by comparison with an appropriate standard (eg, the TLV). This is followed by control measures when the results indicate high exposure levels.^17^

Toluene is collected through activated carbon tubes with specially designed suction pumps that maintain a stable flow throughout the sampling period. Strategies vary to ensure that a representative percentage of the work shift is covered. Passive dosimeters are another type of device equipped with activated carbon to adsorb organic vapors without suction mechanisms. Toluene in air can be analyzed by several techniques, including gas chromatography (GC) and mass spectrometry. The material is desorbed with carbon disulfide, and the most common technique for analyzing toluene is GC with flame ionization detection (GC-FID) according to NIOSH method 1501.^18^

#### Biological monitoring

Biological monitoring is the periodic assessment of occupational exposure by measuring a chemical agent’s concentration in a biological fluid, its biotransformation products, its toxic action, or even expired air as an indicator of exposure. The results are compared to appropriate benchmarks to assess the health risk, with a view to introducing or modifying control measures when necessary.^17^ The studied parameters are called biological indicators, bioindicators, or biomarkers. The method of biological matrix collection, which is based on the representativity of exposure and biotransformation, occurs at the end of the workday or at the beginning or end of the work week.^10^

#### Urinary hippuric acid

Urine is collected at the end of the workday and sent in an amber glass or polyethylene bottle to the laboratory. Although Brazilian legislation has recommended this procedure and, and it is offered by laboratories, it should be discontinued. NIOSH has proposed method 8300 for determining HA-u as a biological indicator of toluene exposure. The analysis matrix is urine, collected at the end of a shift after 2 days of exposure. The volume, which must be 50-100 mL, is transported in a 125 mL plastic bottle. Sample stability is 1 day at 20°C, 1 week at 4°C, and 1 month at -20°C. A control sample must be collected prior to exposure.^4,18^

The analytical technique for this method is absorption spectrophotometry in the visible region, using a wavelength of 410 nm and an optical path of 1 cm. The analyzed compound is a complex of hippuric acid and benzenesulfonyl chloride. The working range should be 0.005 to 0.500 g/L (urine diluted 1:5 v/v). This technique has an estimated detection limit of 0.002 g/L (SD, 0.06%).^18^

To measure joint exposure with xylene or styrene, NIOSH method 8301 for HA-u and methyl hippuric acid is recommended, which involves high performance liquid chromatography with ultraviolet detection.^18^

#### Urinary ortho-cresol

Hasegawa et al.,^19^ who developed the first method for measuring urinary ortho-cresol (ο-cresol) in workers, conducted an important comparative study between urinary HA-u and ο-cresol as bioindicators of toluene exposure. A total of 130 volunteers (74 men) outfitted with passive activated carbon dosimeters were intentionally exposed to toluene in the air over a workday and analyzed with GC-FID. To prepare the sample, 0.5 mL of 15% hydrochloric acid was added to 1 mL of urine and heated at 100°C for 60 min. The samples were hydrolyzed with 3,5-xylenol, and desorption was performed with carbon disulfide. The organic phase was treated with sodium sulfate to dry the sample, which was finally injected into the GC-FID. The results showed that HA-u has a very good correlation with environmental exposure and is reliable at concentrations above 100 ppm (ie, it is inadequate for the current limit of 20 ppm). Below 100 ppm, the results are susceptible to various types of interference. The most important point raised in this study is the method’s applicability to ο-cresol and its good correlation with environmental exposure, being little affected by factors unrelated to environmental exposure to toluene.^19^

Through a partnership between the toxicological analysis departments of the University of São Paulo and the Federal University of Alfenas, a method was developed for determining urinary ο-cresol using solid phase microextraction (SPME) and chromatographic analysis using GC-FID. After optimizing the SPME variables and validating the method, urine samples from workers exposed to solvents were analyzed. The best extraction conditions were obtained with acid hydrolysis of the urine, extraction in carbowax/divinylbenzene fiber 70 µm for 20 minutes at a neutral pH, adding 3 g of Na_2_SO_4_ under agitation. The method showed linearity between 0.1 and 1.5 µg/L of ο-cresol in urine, a quantification limit of 0.1 µg/L, and repeatability between 6.8 and 7.8%. The mean ο-cresol value in worker urine was 0.35 (SD, 0.23) µg/L. The SPME/GC-FID method showed promise, being a quick, simple, and applicable for biologically monitoring workers exposed to toluene.^20^

In 2015, the 5th edition of the NIOSH Analytical Methods Manual presented method 8321 for determining o-cresol in urine. The technique was GC with mass spectrometry and selected ion monitoring. This is the reference method for analyzing urinary ο-cresol in workers exposed to toluene.^21^

#### Other biological indicators

Toluene in exhaled air: samples must be collected during the workday. In 1999, the ACGIH stopped recommending exhaled air as an exposure biomarker due to strong interference from other substances (eg, ethanol) and because it is not representative of skin absorption. GC-FID was the analysis technique, with the sample collected in flasks and transferred to another flask through an activated carbon column. The analysis then proceeded according to NIOSH method 1501.^8^

Blood toluene: samples must be collected before the last shift of the workweek. This biomarker is little used, since the ideal would be to collect several samples during the shift, which, from a practical point of view, is unfeasible and highly invasive. It is still recommended by ACGIH for very specific cases due to its representativeness for skin absorption.^8^ According to NIOSH method 8007, toluene is extracted from the blood by headspace GC and GC-FID is used for identification.^21^

TOL-u: sample collection must occur at the end of the workday. This biomarker has a good correlation with environmental exposure, but it is rarely used due to practical difficulties, such as sample preservation due to the solvent’s volatility. The ACGIH began recommending TOL-u in 2010. The analysis technique involves extracting toluene from urine by headspace GC in association with SPME; GC-FID is used for identification.^22^

## DISCUSSION

Intensive use of toluene over the years has led to adverse health effects in workers, such as central nervous system changes and miscarriages. At the beginning of the 21st century, such critical effects stimulated careful review of occupational exposure to this chemical agent, including a reassessment of tolerance levels. Therefore, understanding the toxicokinetics of toluene has become fundamental, especially regarding respiratory exposure, which is highly significant in occupational safety. Studies are needed are needed to determine biotransformation products that could be used as biological exposure indices, in addition to factors that could interfere with the kinetics.

For most chemical agents, the biological exposure indices are directly correlated with the TLV, and the correlations of some are linked to a certain effect. Even so, the expected results for a biological indicator may not occur due to a number of factors. According to the ACGIH, this is mainly due to the worker’s physiology and health status (eg, age, sex, pregnancy, medication, or diet), factors linked to non-occupational exposure, and lifestyle habits (eg, smoking, and alcohol and drug use). Factors in the work environment must also be taken into account, such as work intensity, air temperature, humidity, the presence of other substances, work hours, and skin exposure.

Despite the many forms of interference, HA-u was recommended in Brazilian legislation until January 2022. Hippuric acid is a normal human metabolite and may originate from diets rich in foods containing benzoic acid and/or or their precursors, especially fruits (prunes, raisins, and peaches) and green coffee beans. Food and drink preserved with benzoates (eg, juice, bread, mustard, soft drinks) increase the formation and excretion of hippuric acid. Soft drinks can produce a concentration of hippuric acid equal to that excreted after occupational exposure of 53 ppm (more than double the current TLV). Several drugs, including cocaine, can increase the physiological excretion of toluene through urine. Benzene and ethanol interfere with the biotransformation of toluene and, consequently, with excretion of its metabolites derived from benzoic acid. In addition, HA-u significantly differs from baseline exposure values only above 30 ppm of toluene in the air, which is 50% higher than the current TLV. This is perhaps the most relevant point in the discussion of ο-cresol as a bioindicator of toluene.^9^

Toluene in exhaled air is a biomarker that correlates well with occupational exposure, although it is subject to interference (eg, ethanol) and is not representative of skin exposure. TOL-u has some advantages, such as a good correlation with exposure and the fact that it does not require correlation with creatinine or sample density, but it has a low urinary concentration and the solvent may volatilize in the sample between collection and analysis. Blood toluene involves a problem that makes it practically unfeasible: the rapid passage of toluene into richly vascularized and soft tissues makes it unrepresentative of an 8-hour workday.

Urinary ο-cresol, although recommended by the ACGIH since 2007, was only included in Brazilian legislation in 2020, becoming effective in 2022. Compared to HA-u, this biomarker is advantageous because few factors interfere in it and it is virtually absent from the urine of unexposed persons. Its main disadvantage is that a very small fraction of toluene is transformed into ο-cresol, since it uses a different biotransformation pathway from hippuric acid and might not be detected after very light exposure. Thus, for ο-cresol, the technique and analytical method must have high sensitivity, which is not really a limiting factor, considering the current analytical instrumentation.

While the discussion revolves around a biomarker with a better “cost/benefit” ratio in terms of specificity/sensitivity/correlation for a 20 ppm TLV, and even considering future validation of S-benzylmercapturic and S-p-toluylmercapturic acids as biomarkers for toluene, it appears that urinary ο-cresol is the only practicable one.

## CONCLUSIONS

Institutions and researchers involved in TLV and biological indicator studies report the difficulty of establishing parameters that are safe for occupational exposure to chemical agents. In addition to the wide dose-response curve of the studied populations, the multifunctionality of modern workers, and the lack of more in-depth studies on new substances, we must also consider political-economic issues that, in practice, have a strong influence on chemical agent use and the protection of worker health.

Regarding analytical issues, the focus should be on the representativity of tolerance limits, bioindicators, and other factors in the work environment to produce a faithful estimate of worker exposure to the chemical agent. The sensitivity of the method must meet the tolerance limits, and the biological indicator must be specific enough not to suffer interference from food or concomitant exposure to other substances or interactions. In this respect, it has been proven that HA-u is no longer useful as a biological indicator of toluene, despite still being used in Brazil.

Many studies over the last three decades have demonstrated that urinary ο-cresol has all the characteristics of a good biological indicator for toluene, especially with the new lower TLV of 20 ppm. Until recently, professionals used HA-u, arguing that the then-current legislation considered a tolerance limit of 78 ppm. However, revision of Regulatory Norm 7 and the alignment of bioindicators with international recommendations have brought order to this discussion, which has been ongoing in Brazil for more than a decade. In January 2022, the new Regulatory Norm 7 came into force, establishing urinary ο-cresol, blood toluene, and TOL-u as bioindicators for toluene. Given the scientific evidence, it is expected that urinary ο-cresol will be the first choice in occupational medicine.
